# Oculomotor Deficits in Aryl Hydrocarbon Receptor Null Mouse

**DOI:** 10.1371/journal.pone.0053520

**Published:** 2013-01-03

**Authors:** Aline Chevallier, Antoine Mialot, Jean-Maurice Petit, Pedro Fernandez-Salguero, Robert Barouki, Xavier Coumoul, Mathieu Beraneck

**Affiliations:** 1 INSERM UMR-S 747, Toxicologie Pharmacologie et Signalisation Cellulaire, Centre universitaire des Saints-Pères, Paris, France; 2 Centre d'Etude de la Sensori Motricité - CNRS UMR 8194, Centre universitaire des Saints-Pères, Paris, France; 3 Assistance Publique-Hôpitaux de Paris, Hôpital Necker-Enfants Malades, Paris, France; 4 Universidad de Extramadura, Extramadura, Spain; 5 Université Paris Descartes, Sorbonne Paris Cité, Paris, France; University of Medicine & Dentistry of NJ - New Jersey Medical School, United States of America

## Abstract

The Aryl hydrocarbon Receptor or AhR, a ligand-activated transcription factor, is known to mediate the toxic and carcinogenic effects of various environmental pollutants such as 2,3,7,8-Tetrachlorodibenzo*-p-*dioxin (TCDD). Recent studies in *Caenorhabditis elegans* and *Drosophila melanogaster* show that the orthologs of the AhR are expressed exclusively in certain types of neurons and are implicated in the development and the homeostasis of the central nervous system. While physiological roles of the AhR were demonstrated in the mammalian heart, liver and gametogenesis, its ontogenic expression and putative neural functions remain elusive. Here, we report that the constitutive absence of the AhR in adult mice (AhR−/−) leads to abnormal eye movements in the form of a spontaneous pendular horizontal nystagmus. To determine if the nystagmus is of vestibular, visual, or cerebellar origin, gaze stabilizing reflexes, namely vestibulo-ocular and optokinetic reflexes (VOR and OKR), were investigated. The OKR is less effective in the AhR−/− mice suggesting a deficit in the visuo-motor circuitry, while the VOR is mildly affected. Furthermore, the AhR is expressedin the retinal ganglion cells during the development, however electroretinograms revealed no impairment of retinal cell function. The structure of the cerebellum of the AhR−/− mice is normal which is compatible with the preserved VOR adaptation, a plastic process dependent on cerebellar integrity. Finally, intoxication with TCDD of control adults did not lead to any abnormality of the oculomotor control. These results demonstrate that the absence of the AhR leads to acquired central nervous system deficits in the adults. Given the many common features between both AhR mouse and human infantile nystagmus syndromes, the AhR−/− mice might give insights into the developmental mechanisms which lead to congenital eye disorders.

## Introduction

The Aryl hydrocarbon Receptor (AhR) is a transcription factor known to mediate most of the toxic effects of widely persistent organic pollutants such as dioxin (TCDD; 2,3,7,8 TetraChloroDibenzo-p-Dioxin). This receptor belongs to the basic helix-loop-helix Per/ARNT/Sim family (bHLH-PAS) whose members are involved in physiological processes such as circadian cycle, neurogenesis and organs development [1]. Historically, the AhR has been described as a ubiquitous xenobiotic-activated transcription factor, which promotes the elimination of xenobiotics through the regulation of the expression of genes involved in xenobiotic metabolism [2].

Interestingly, recent studies suggest that this receptor also regulates alternative signaling pathways independently of exposure to pollutants. AhR-null mice exhibit infertility, abnormalities in liver and cardiovascular problems such as a defective closure of the ductus venosus, indicative of the implication of the AhR in various developmental processes [3], [4], [5].

In the rodent brain, the AhR mRNA was reported to be expressed in the cerebral cortex [6], hypothalamus and brainstem [7] and in the granular cells of the cerebellum [8]. However, its putative endogenous neuronal functions in mammals remain unknown. Interestingly, invertebrates express an AhR ortholog (AhR-1 in *Caenorhabditis elegans* and Spineless in *Drosophila melanogaster*) in neurons: Spineless is expressed in the sensory neurons of the peripheral nervous system and AhR-1 is expressed in several subtypes of neurons including touch receptors and interneurons. This protein however does not bind dioxin, one of the most potent ligand and activator of the mammalian AhR [9]. Analysis of AhR-1 mutants shows that, in *C*. *elegans*, the receptor is implicated in GABAergic neuron cell fate [10]. Recent investigations using *Drosophila melanogaster* as a model, have confirmed the importance of AhR orthologs for the neuronal organization in invertebrates: the Spineless gene was initially identified as a critical gene for the proper specification of distal antennal identity, the establishment of the tarsal regions of the legs, and a normal bristle growth [11]. In addition, one recent report implicates Spineless in the regulation of dendritic arborization of sensory neurons [12].The strong conservation of the AhR during species evolution [13] and its ancestral function in invertebrates suggest that in mammals, the AhR could play a significant role in the development and/or the homeostasis of the central nervous system (CNS). So far, only one study showed a reduction of the DNA content and expression of a GABAergic receptor, GABAR-Aα6, in the cerebellum of the AhR−/− mice [8]. However, the potential endogenous role of the AhR in the mammalian brain remains elusive.

In this context, we investigated the role of the AhR in the nervous system of mice. We monitored spontaneous and reflexive eye movements to assess the integrity of the gaze-related sensorimotor functions in the AhR−/− mice and identified a pendular horizontal nystagmus in those animals. The basic gaze stabilizing reflexes, namely the vestibulo-ocular and optokinetic reflexes, and the adaptative capacities of the cerebellum were then evaluated.

## Materials and Methods

### Ethics Statement, Animals and Treatment

All procedures used were in strict compliance with the European Directive 86/609/EEC on the protection of animals used for experimental purposes. CNRS review board and the Direction Départementale des Services Vétérinaires approved this study; authorization number 75-1641 and 75-962. AhR−/− (C57BL/6J background) and AhR +/+ (Wild-Type, C57BL6/J) mice are a generous gift of Pr. PM Fernandez-Salguero [3]. Twenty-one AhR+/+ mice, thirteen AhR+/− mice and fifteen AhR−/− mice were used in this study. In addition, eight male mice (10 weeks) were injected i.p. with 200 µL of TCDD (25 µg per kg of body weight) or the vehicle (corn oil) (four mice/group). The chronic treatment consisted of one injection of TCDD each week during 5 weeks (total of 5 injections).

### Eye Movements Recording

Male AhR−/−, AhR+/− and AhR+/+ mice aged of 10 weeks, were included in these tests (n = 9 for AhR+/+, n = 8 for AhR+/− and n = 13 for AhR−/− mice). Surgical preparation and postoperative care for head implant surgery have been described previously [14]. Briefly, gas anaesthesia was induced using isoflurane. The head was shaved using an electric razor. A sagittal incision of about 2 cm was performed to access the skull. Then, a small custom-built head holder (0.3×0.3×0.5 cm) was fixed using dental cement (C&B Metabond; Parkellinc, Edgewood, NY) to the skull just anterior to the lambda landmark [15]. Following the surgery, animals were isolated and closely surveyed for 48 hours. Buprenorphine (0.05 mg/kg) was provided for postoperative analgesia and care was taken to avoid hypothermia and dehydration.

The experimental set-up, apparatus, and method of data acquisition used to record eye movements were similar to those previously described [14], [16]. Mice were head-fixed at a ∼30° nose-down position to align the horizontal semi-circular canals on yaw plane [14], [15], [17] and then placed in a custom built Plexiglas tube secured on the superstructure of a vestibular stimulator. Eye movements were tracked using an infrared video system (ETL-200, ISCAN, Burlington MA). Recorded eye and head (table) position signals were sampled at 1 kHz, digitally recorded (CED power1401 MkII) under Spike 2 environment and later exported into the Matlab (The MathWorks) programming environment for off-line analysis.

Stimulation protocol: light intensity in the experimental room was of 350 Lux (Luxmeter Lux-1337 Iso-tech). Light condition was used to record spontaneous eye movements, during visuo-vestibular conflict and for the optokinetic reflex. For tests performed in dark (spontaneous eye movements and VOR) all sources of light were turned off except for computer screen. The turntable is further surrounded with a closed box to isolate the animal from remaining light. “Dark” condition inside the box was measured as <0.02lux. The different tests were performed as follows: i) As eyes were recorded both at light and in the absence of light, 2% pilocarpine (Laboratoire Chauvin) was applied 10 minutes before to start the experiment to keep the pupil size constant [18], [19]; ii) Spontaneous eye movements were first recorded in the absence of external stimulation at light (350lux) and then in dark (<0.02lux); iii) Vestibulo-ocular reflex in dark was tested during sinusoidal rotation of the turntable (at frequencies of 0.2; 0.5; 1 and 2 Hz performed at a constant peak velocity of 50°/s); iv) Optokinetic full field stimulation was performed at light by rotating the animal at a constant velocity of 5°/s. In the mouse, optokinetic speed tuning curve for the eye movement responses demonstrated peak gains at velocities of rotation in range 0–5°/s (Stahl et al. 2004; see figure 8A in Beraneck and Cullen, 2007); v) in a separate session, the turntable was surrounded by a box with highly contrasted pattern and no ceiling. Visuo-vestibular conflict (VVC) was then performed in light condition by rotating sinusoidally the animal en-bloc with the visual surround at 0.5 Hz (i.e. table and visual surround are moving in the same direction; peak velocity 50°/s). Training sessions lasted 45 min and 2 min-long eye movement recordings were performed every 15 min. Vestibulo-ocular reflex in dark was also recorded just before and right after each VVC training paradigm.

### Data Analysis

Analysis procedures for horizontal angular vestibulo-ocular reflex (VOR) and optokinetic reflex (OKR) have already been reported elsewhere [14]. Horizontal and vertical eye and head movements data were digitally low pass-filtered (cut-off frequency: 40 Hz), and position data were differentiated to obtain velocity traces. Segments of data with saccades were excluded from analysis. For horizontal sinusoidal rotations, at least 10 cycles were analyzed for each frequency. VOR gain and phase were determined by the least-squares optimization of the equation:
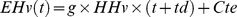
Where EHv(t) is eye horizontal velocity, g (gain) is constant value, HHv (t) is head horizontal velocity, td is the dynamic lag time (in msec) of the eye movement with respect to the head movement, and C^te^ is an offset. td was used to calculate the corresponding phase (φ°) of eye velocity relative to head velocity. The Variance-Accounted-For (VAF) of each fit was computed as:



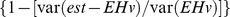
Where *var* represents variance, *est* represents the modeled eye velocity, and *EHv* represents the actual eye horizontal velocity. VAF values were typically between 0.70–1, where a VAF of 1 indicates a perfect fit to the data. Trials for which the VAF was less than 0.5 were excluded from the analysis.

Because AhR−/− mice show spontaneous nystagmus, OKR responses were measured as the mean eye velocities on segments longer than 2/F_n_ where F_n_ is the measured frequency of the spontaneous nystagmus. OKR gains were then calculated as the ratios of the mean eye velocities to the constant table velocity.

### Immunohistochemistry

Mice were perfused transcardially with 4% PFA, the brain was removed and post-fixed for 3 h, and then cryoprotected in 10% sucrose. Sagittal cerebellum sections of 14 µm were cut using cryostat (Leica CM 3050S). Sections were blocked in 10% Normal Goat Serum (NGS, Invitrogen) in PBS containing 1% triton X-100 (Sigma-Aldrich) and incubated overnight at 4°C with primary antibodies mouse anti-CaBP (1∶400, Swant) or guinea pig anti-VGLUT2 (1∶1000, Millipore). After several washes, sections were incubated with secondary antibodies Alexa 488 Goat anti-mouse (1∶400, Invitrogen) and Goat anti-guinea pig and Alexa 546 Goat anti-mouse (1∶400, Invitrogen). Eyes of mouse were enucleated, post-fixed for 3 h, and then cryoprotected in 10% sucrose for 1 h at room temperature and in 30% sucrose overnight at 4°C. The samples were embedded in OCT solution (VWR) and frozen in isopentane. Sections of 20 µm were cut using cryostat (Leica CM 3050S), blocked in PBS containing 0.2% of gelatin and 0.25% of Triton X-100 for 1 h at room temperature and incubated with primary antibodies CaBP (1∶500, Swant), Calretinin (1∶500, Millipore), PKCα (1∶500, Sigma), α-Recoverin (1∶1000, Millipore). After washing, slides were incubated with secondary antibodies Alexa 488 chicken anti-rabbit (1∶400, Invitrogen) or TRITC goat anti-mouse (1∶100, Jackson ImmunoResearch). The number of retinal ganglion cells was assessed on flat mount retinal preparation after immunohistochemistry using ßIII-tubulin (1∶500, Covance). Eight adult male mice (n = 4 AhR+/+ and n = 4 AhR−/−) were used and five pictures of each staining were analyzed for the quantification.

The sections were, then, counterstained with DAPI (1∶2000, Invitrogen) and examined under an epifluorescence microscope (Nikon Eclipse TE-2000E) and confocal microscope (Zeiss, LSM 710). The density of retina cells and the thickness of the layer is measured using imageJ software.

### *In situ* Hybridization

AhR probes were generated using cDNA from the mouse brain. A 1,2 kb fragment of AhR (nt: 1622–2828 bp) was subcloned in pbluescriptKS+. Embryons (E12 and E14) of AhR+/+ and AhR−/− mice were removed and post-fixated during 24 h in a solution containing 4% paraformaldehyde in 0.12 M phosphate buffer, pH 7.4 (PFA), cryoprotected in sucrose 10% O/N and frozen in isopentane. Coronal sections of 14 µm were cut with a cryostat (Leica CM 3050S). Sections were mounted on superfrost gold plus slides and store at −80°C until use. Tissue sections were hybridized with digoxigenin-labeled riboprobes. Tissue sections were post-fixed for 10 min in 4% PFA, washed in PBS, pH 7.4, treated with proteinase K (5 µg/mL; Invitrogen, Carlsbad, CA) for 2 min, post-fixed for 5 min in 4% PFA, washed in PBS, acetylated, and washed in PBS 1% Triton X-100. Slides were incubated for 2 hr at room temperature in hybridization buffer (50% formamide, 5X SSC, 1X Denhardt's, 240 µg/mL yeast tRNA, and 500 µg/mL DNA salmon sperm), and then tissue sections were hybridized overnight at 72°C with riboprobes (0.5 ng/µL). After hybridization, sections were rinsed for 2 hr in 2× SSC at 72°C and blocked in 0.1 MTris, pH 7.6, 0.15 M NaCl, 0.1% Tween-20 (B1) containing 10% normal goat serum (NGS) for 1 hr at room temperature. Slides were incubated overnight at 4°C with anti-digoxigenin antibody conjugated with the alkaline phosphatase (1∶5000; Roche Diagnostics) in B1 containing 1% NGS. After washes, the alkaline phosphatase activity was detected using nitrobluetetrazolium chloride (NBT) (337.5 µg/mL) and 5-bromo-4-chloro-3-indolyl phosphate (BCIP) (175 µg/mL) (Roche Diagnostics). Sections were stained with DAPI (Invitrogen) and mounted in Mowiol (Calbiochem/Merck, Carlstadt, Germany). Control sense probe yielded no signal.

### Electroretinograms (ERGs)

ERGs were recorded from male AhR−/− (n = 8) and AhR+/+ mice (n = 6) aged of eight weeks. Mice were dark-adapted overnight and prepared under red dim light. Mice were anesthetized with intraperitoneal injection of ketamine 500 (100 mg/kg, Virbac, France) and xylazine (10 mg/kg, Rompun 2%, Bayer). Pupils were dilated with tropicamide eye drops (Mydriaticum 0.5%, Théa, France) and anesthesized by oxybuprocain hydrochloride solution (Théa, France) applied prior to the recordings. ERGs were recorded from both eyes simultaneously. The electroretinography equipment (SIEM Bio-Médicale, France) consisted of a Ganzfeld bowl, an amplifier (0.1 to 300 Hz) and a PC-based control (VisioSystem). Single-flash responses were obtained under dark-adaptated (scotopic) and light-adaptated (photopic) conditions. For the scotopic condition, five responses were averaged per light intensity with an inter-stimulus interval of 30s. Increasing white-flash stimuli ranged from −2 to −0.49 log cd.s/m^2^. For the photopic condition, light adaptation was performed with a uniform background light (−1.52 log cd.s/m^2^) during 5 minutes and ten responses of white-flash of −0.49 log cd.s/m2 were averaged.

### Statistical Analysis

Statistical processing of all results was carried out using the Statistica 7.1 software (StatSoft France). For all behavioural tests, we performed a two-way repeated measure ANOVA followed by a post-hoc Tukey analysis. For quantification of retina cells, a Friedman test was performed. The threshold for significance was set at p<0.05. Reported numbers and figure error bars represent ± standard deviations (SD).

## Results

### The AhR−/− Mice Suffer from a Nystagmus-like Ocular Instability

**Figure 1:**
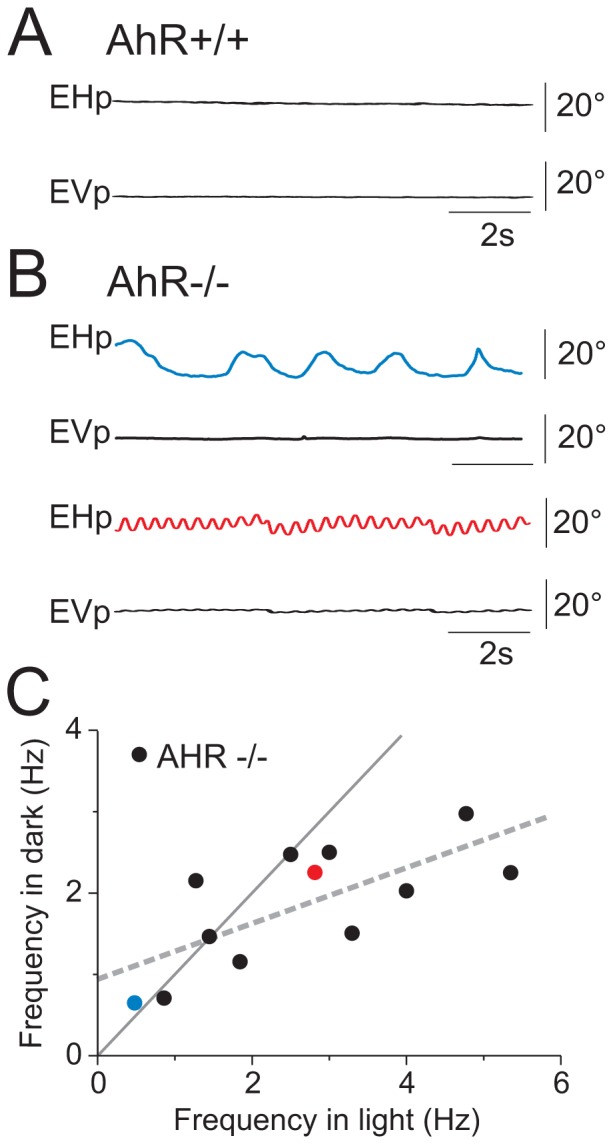
AhR−/−mice have a horizontal pendular nystagmus. (A) Positions of the eyes of the AhR+/+ mice in both horizontal (EHp) and vertical (EVp) plans in the absence of head movements in the dark. (B) Positions of the eyes of 2 different AhR−/− mice showing spontaneous nystagmus at low frequency in light (upper) and at high frequency in dark (bottom). The AhR−/− mice have an ocular instability exclusively in the horizontal plan whereas the eyes of AhR+/+ mice and AhR+/− mice are stable. (C) Frequencies of the nystagmus in light and dark conditions for each AhR−/− mouse (n = 12). The linear regression is represented by the dotted line in grey. EHp: Eye Horizontal position; EVp: Eye vertical position. In this and all following figures, eye movements to the right are presented upward.

Because a great deal is known about the anatomy and physiology of the circuits responsible for gaze control, spontaneous and reflexive eye movements can be used to assess the integrity of many sensorimotor functions [20]. Thus, to assess potential neurological brain dysfunctions, spontaneous and reflexive eye movements were monitored in the AhR−/− mice. First, video-oculography was performed in dark [14]. Figure 1A presents the eye position in horizontal and in vertical plans in the absence of head movements (spontaneous eye movements). As animals are quietly seated in dark, the eyes of wild-type mice (AhR+/+) and heterozygous mice (AhR+/−, not shown) remain stable. In contrast, the gaze of AhR−/− mice is unstable at rest, as revealed by the presence of a spontaneous horizontal movement observed in all animals (n = 12) (Figure 1B). This ocular instability affects both eyes and is also observed in the light. It consists in rhythmic sinusoidal, purely horizontal eye movements. The frequency of the ocular instability varies from 0.5 to 5 Hz depending on each individual AhR−/− mouse (mean in dark = 1.84±0.74 Hz; figure 1B with two examples: 0.5 Hz, upper panel to 2.8 Hz, lower panel; note absence of vertical movement) and varies with the lighting condition: indeed, we systematically measured the frequency of the eye oscillation for each mice (n = 12) in the dark and in the light (Fig. 1C). For a majority of individuals (n = 8; equation: y = 0.94+0.34x, r^2^ = 0.5089; ANCOVA F(1,22) = 9.41, p<0.01), the frequency of the nystagmus in the light is higher. This last observation is commonly observed with congenital nystagmus in humans, as the nystagmus increases when the patient tries to fixate an object.

We also performed the experiment with female mice (n = 4) and identified a horizontal pendular nystagmus, similar to the one reported in males (not shown); the deficit is therefore not gender related. In addition, recordings obtained with 2 males tested at different ages (Figure S1) suggested that the nystagmus is present and evolves throughout the lifespan of the animals (pendular nystagmus to jerk nystagmus). Overall, the presence of a spontaneous nystagmus in mice of different ages and gender, demonstrates that the AhR−/− mice consistently suffer from impairment in gaze control.

### Assessment of the Vestibulo-ocular Reflex

**Figure 2:**
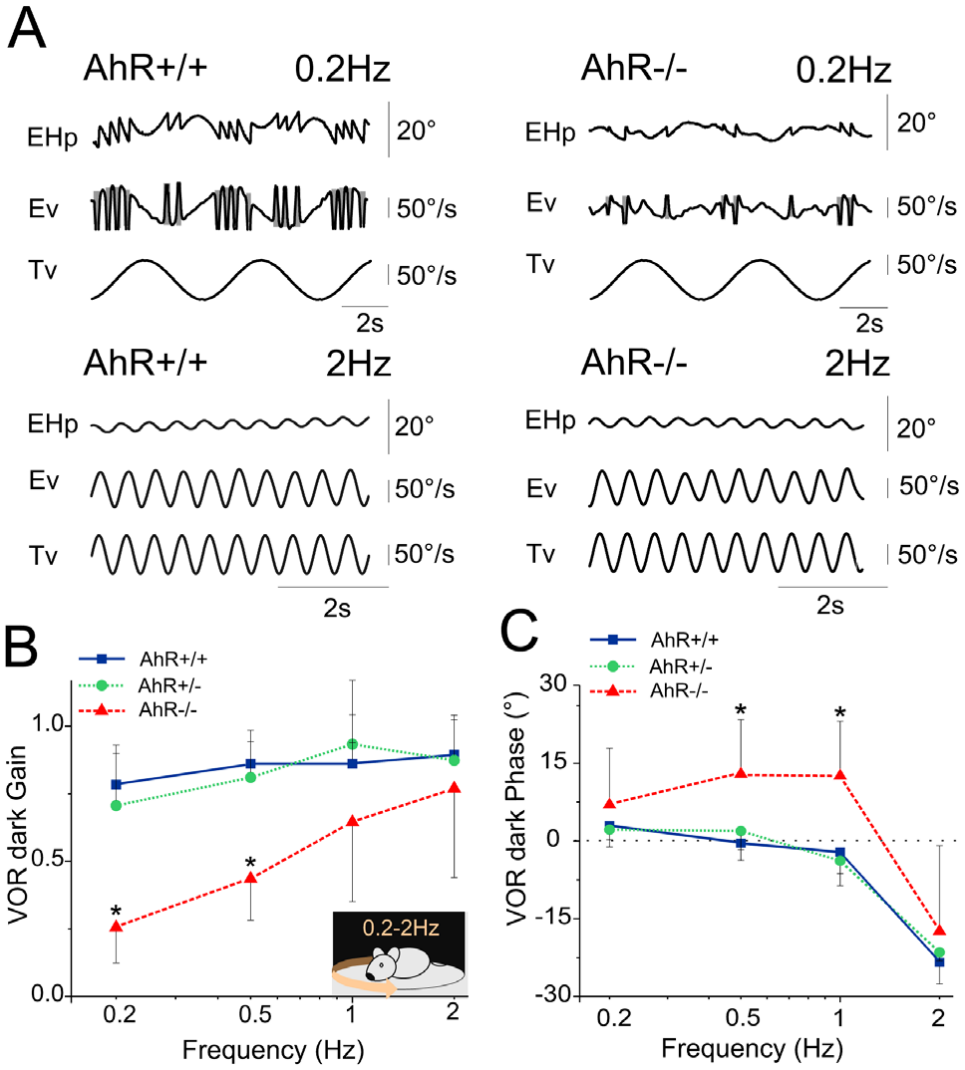
Normal vestibular function in AhR−/− mice. (A) Example of eye movements evoked in AhR+/+ (*left*) and AhR−/− (*right*) during sinusoidal oscillation in the horizontal plan at a peak velocity of 50°/s. *top*: frequency of the oscillation equal to 0.2 Hz; *bottom*: frequency of 2 Hz. Shaded areas indicate quick phases. At low frequency, the nystagmus overlaps the normal VOR pattern. At high frequency, both AhR+/+ and AhR−/− mice show comparable reflexive eye movements. (B–C), Gain (B) and phase (C) of the VOR in the dark. Blue lines correspond to the AhR+/+ mice, green lines correspond to the AhR+/− mice and red lines correspond to the AhR−/− mice. At low frequency, the VOR gain and phase in the AhR−/− mice decreased compared to the AhR +/+ mice. At high frequency, VOR gain and phase did not differ from that of other groups suggesting that the vestibulo-ocular reflex is not affected in the AhR−/− mice. Asterisk indicates statistical difference with *p*<0.05, EHp, Eye Horizontal position; EVp, Eye Vertical position; Ev, Eye velocity; Tv, Table velocity. In this and all following figures: head velocity traces have been inverted to facilitate comparison with eye velocities; the phase lead is positive (up); plots present mean ± Standard Deviation (SD).

Typically, spontaneous nystagmus can be of visual and/or vestibular origins. To explore the putative vestibular origin of the pendular nystagmus, we tested the functional integrity of the vestibulo-ocular reflex (VOR) of the AhR−/− mice (Figure 2). Sinusoidal rotations of AhR+/+ (Figure 2A, left panel) and AhR+/− mice (not shown) in the dark leads to a typical stabilization of the eye movements as compensatory slow phases interrupted by quick-phases that re-center the eye in the orbit (Figure 2A). In contrast, in AhR−/− mice (Figure 2A, right panel), the spontaneous nystagmus overlaps with the former described pattern (Figure 2A, upper panel). Interestingly, at higher frequencies (above 1 Hz), the vestibular reflex dominates the spontaneous instability of the eye, which leads to a response qualitatively equivalent to the one measured in the AhR+/+ mice (Figure 2A, left and lower panel).

It should be noted that for the AhR−/−, the nystagmus frequency is in range or close to the range of the table frequencies tested, which makes it impossible to discriminate if the eye movement is caused by the nystagmus or in response to the head movement. We measured the amplitude (gain, i.e the ratio between the eye velocity and the head stimulation velocity, see methods) and timing (phase, i.e the synchrony between the head movement and the reflexive eye velocity, see methods) of the eye movement evoked during rotation of the table. At low frequencies (0.2 and 0.5 Hz), the VOR dark gain of the AhR−/− is significantly lower than in controls, most probably because at these intensities, the nystagmus largely dominates the reflexive eye movements (F(2,25) = 10.7, p<0.001) (Figure 2B). The phase of the reflex was also found abnormal at 0.5 and 1 Hz in the AhR−/− mice (Figure 2C) with also a significant effect of the genotype (F(2,25) = 17.47, p<0.001). Those defects were not apparent at higher frequencies (2 Hz), where both amplitude and phase were normal compared to controls. Overall, gaze stabilization was therefore found to be impaired at frequencies up to 1 Hz.

These observations indicate that the congenital nystagmus observed in AhR−/− mice impaired gaze stabilization as long as the head movement was of mild intensity; however, the vestibular stimulation triggered by head movements of higher intensity led to a normal compensatory eye movement and a proper gaze stabilization. Our interpretation is that despite the obvious impairment in gaze stabilization caused by the nystagmus, the 3 neurons-arc which constitutes the basic circuitry underlying the vestibulo-ocular reflex is functional and probably not directly affected by the AhR defect. Together with the absence of obvious deficits in postural control (data not shown), these results suggest that the nystagmus of AhR−/− mice is unlikely to be of purely vestibular origin.

### Assessment of Cerebellum Integrity and Function

**Figure 3:**
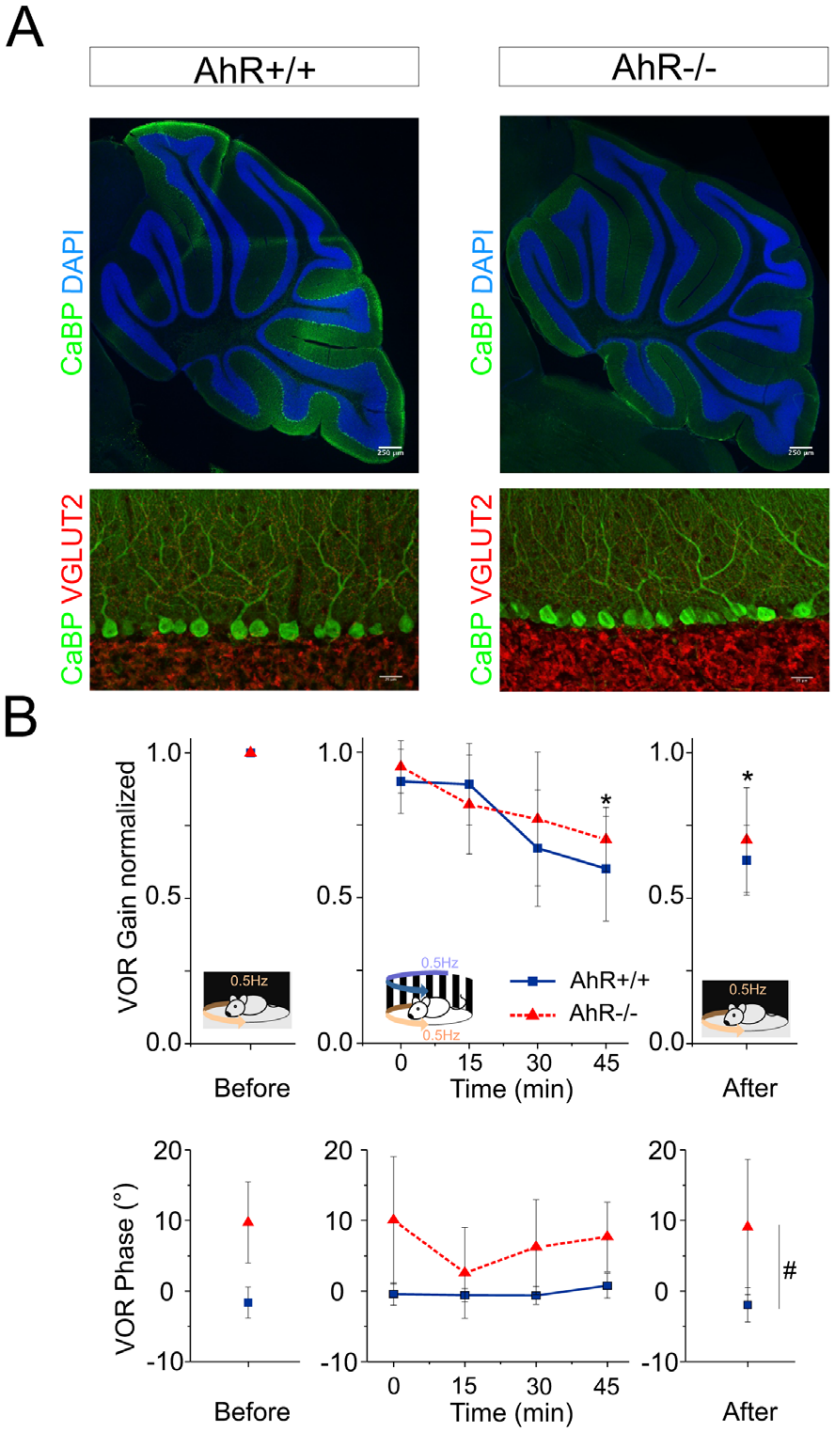
Cerebellar morphology and function are not affected by the AhR invalidation. (A) The morphology of the cerebellum in both AhR+/+ (*left*) and AhR−/−mice (*right*) is similar. *Top:* The size of the cerebellum and its foliation are normal in AhR−/−, as shown on sagittal sections labeled with anti-calbindin antibodies and counterstained with DAPI. *Bottom:* Climbing fibers (stained by VGLUT2) properly innervate the dendritic arborization of Purkinje cells (CaBP). Scale bars represent 250 µm (*top*) and 25 µm (*bottom*). (B–C) Normalized gain (C) and phase (D) during the visuo-vestibular conflict at 0.5 Hz. The AhR−/− and AhR+/+ mice are capable of adaptation during and after the vestibulo-ocular conflict. Blue lines correspond to the AhR+/+ mice, and red lines correspond to the AhR−/− mice. Asterisk indicates statistical difference between the VOR gain before and after the conflict with *p*<0.001, # represent the statistical difference between the VOR phase during all the adaptation protocol with p<0.001.

The cerebellum plays a central role in gaze stabilizing reflexes, and in particular is part of the pathway involved in the OKR and in the tuning of the VOR. Cerebellar deficits are commonly accompanied by nystagmus [21], [22]. In addition, AhR mRNA was reported in the cerebellum of mammals [23]. We therefore explored the integrity and the function of the cerebellum in the AhR−/− mice. First, the overall cerebellar morphology in the AhR−/− mice was characterized. CaBP staining reveals similar cerebellar morphologies in the AhR−/− mice compared to AhR+/+ mice (Figure 3A). In both cases, the cerebellum has a normal foliation pattern and cytoarchitecture: the Purkinje cells (characterized by CaBP staining) are organized in regular rows and the climbing fibers (characterized by VGLUT2 staining) properly innervate the Purkinje cells (Figure 3A).

While being normally structured, the cerebellum might still present defective functions. In response to a visuo-vestibular conflict, the cerebellum is known to play a central role in the gain-down adaptation of the VOR [24]. Figure 3B shows that the amplitude of the 0.5 Hz VOR of AhR+/+ mice is adapted while the animals are exposed to the visual-vestibular conflict, from 0.90±0.06 to 0.60±0.09 after 45 minutes of adaptation. In the AhR−/− mice, the VOR gain also significantly decreases from 0.95±0.04 down to 0.70±0.04 (adaptation: F(4,36) = 8.4537, p<0.001). In all conditions, the VOR phase is however significantly different between both AhR+/+ and AhR−/− mice (F(1,9) = 34.109, p<0.001), but this difference relates to the genotype, and is not specific to the adaptation. These results suggest that the AhR−/− mice are capable of adaptation and rule out major functional cerebellum-related deficit.

### Assessment of the Optokinetic Reflex

**Figure 4:**
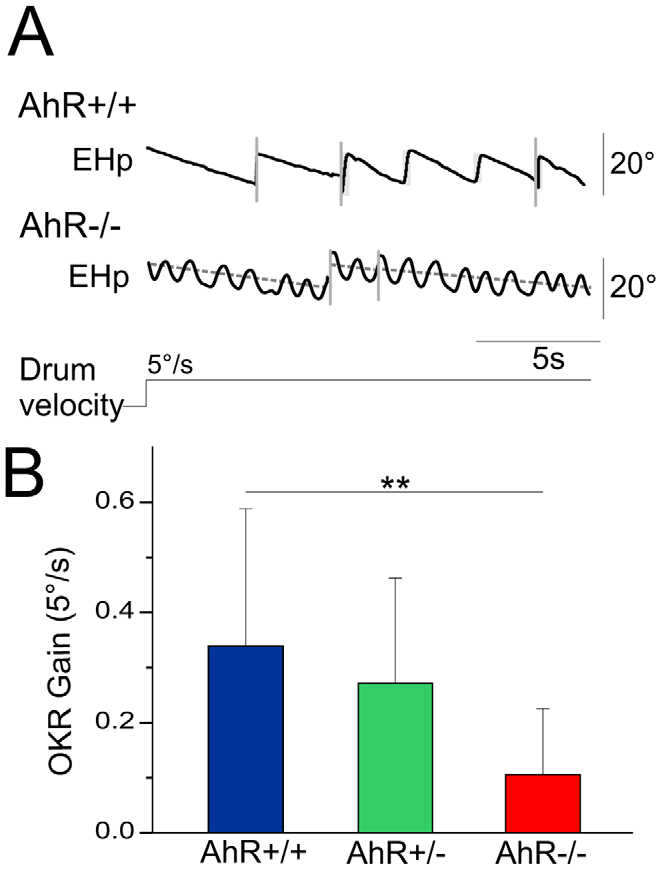
The OKR of AhR−/− mice is decreased. (A) Example of eye movements evoked in both AhR+/+ and AhR−/− mice in horizontal position (EHp) during a rotation at 5°/sec in light in counter-clockwise. (B) OKR gain at 5°/sec. The OKR gain is significantly decreased in the AhR−/− mice compared with AhR+/+ mice. The figure presents discontinuous time, as indicated by the vertical lines. Asterisk indicates statistical difference with p<0.01, EHp: Eye Horizontal position.

To assess the integrity of the visual pathway, we then tested the optokinetic reflex (OKR), a purely visual gaze-stabilizing reflex, by rotating the mouse at a constant velocity (5°/sec) in the light (full field OKR). The recording of the eye movements of both AhR+/+ and AhR−/− mice is illustrated in the figure 4A. In response to optokinetic stimulation, the recording of the horizontal eye movements of the AhR+/+ mice (similar to the AhR+/− mice) shows a classical pattern of slow phases all directed in compensatory direction (same as visual stimulation). In contrast, the pattern recorded in the AhR−/− mice is perturbed by the presence of the spontaneous nystagmus superimposed to the optokinetic response.

We then quantified the gain of the OKR by measuring the dominant eye velocity trend (see methods, dotted line represented in figure 4A). Figure 4B shows for the AhR−/− mice, that it is significantly decreased compared to AhR+/+ controls (F(1,36) = 11.362, p<0.01), which suggests that the OKR circuitry is affected by the mutation.

In conclusion, functional tests of gaze stabilizing reflex suggest that the nystagmus observed in AhR−/− mice would be compatible with the presence of visual or visuo-motor defects, while it unlikely reflects vestibular-specific or cerebellar-specific deficits. The cellular basis underlying the nystagmus is further addressed in the discussion.

### A Role for the AhR in Retina during Development

**Figure 5:**
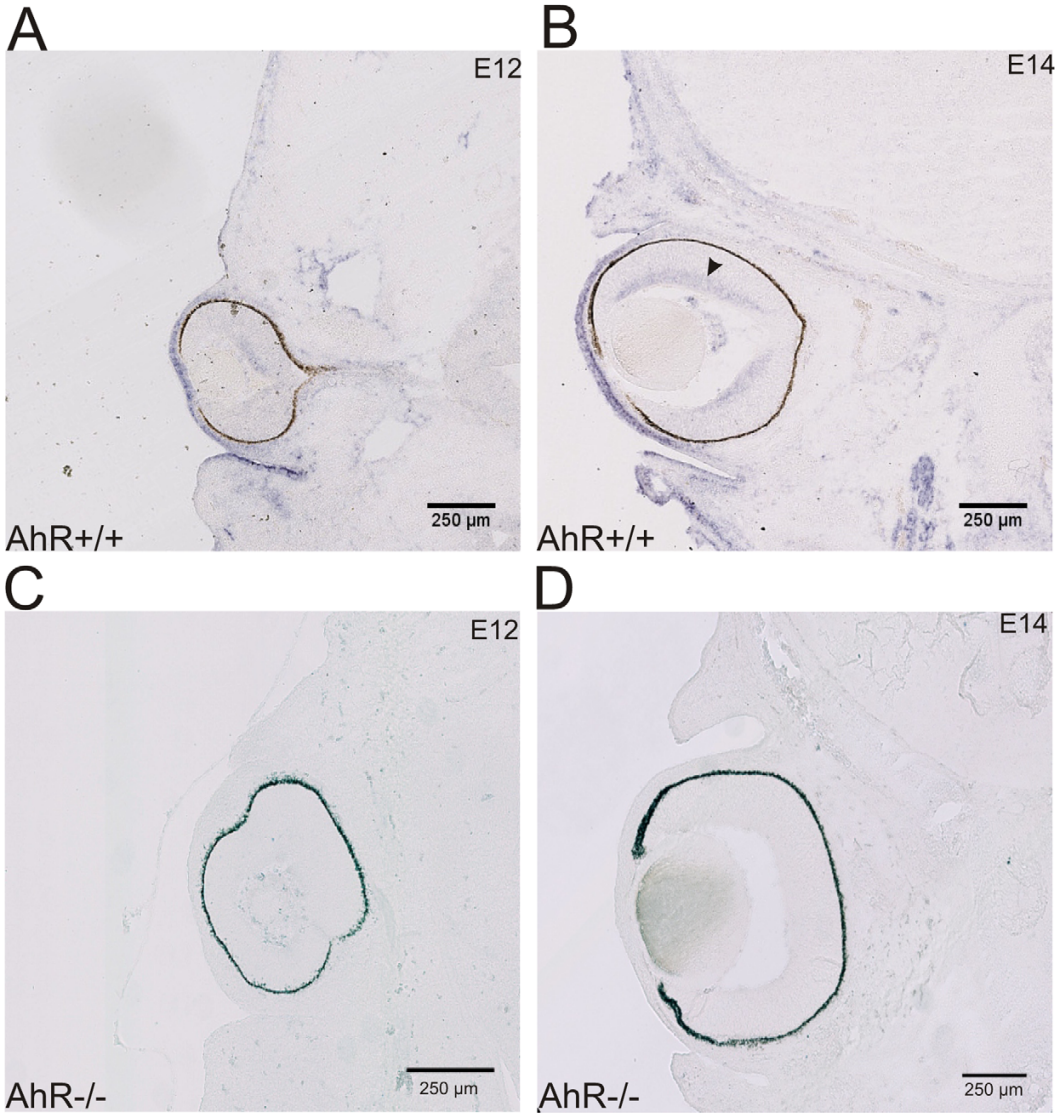
The AhR is expressed in the eyes during the development of AhR+/+ mice. *In situ* hybridization is performed on coronal sections of the mice eyes at two embryonic stages (E12 and E14) with digoxigenin-labeled riboprobes for AhR. (A & C) E12 coronal section of AhR+/+ (A) and AhR−/− mice (C); no expression of AhR is detected at this stage. (B & D) E14 coronal section of AhR+/+ (B) and AhR−/− mice (D); the black arrow (in B) indicates the expression of AhR in the retinal ganglion cells. Scale bar represent 250 µm.

The presence of a congenital nystagmus in the AhR−/− mice and the deficit in the OKR reflex suggest that the AhR might play a developmental function in the visuo-motor system. Therefore, *in situ* hybridization was performed to characterize the expression of the AhR in wild-type embryos at 12 and 14 days of development (Figure 5A: E12; figure 5B: E14) and focused on the eye, brainstem and cerebellum where most of OKR-related structures are found. Interestingly, the AhR is expressed in the retinal ganglion cells (RGCs) at E14, but not present at E12. On the other hand, no trace of the AhR mRNA was found in the brainstem oculomotor-related nuclei or in the cerebellum (data not shown). In the AhR−/− mice, no expression of AhR was detected (Figure 5C: E12; figure 5D: E14). The developmental expression of the AhR in the sensory cells responsible for the OKR would be compatible with the presence of a congenital nystagmus of visual origin. Together with the influence of light on the nystagmus and with the decrease in the OKR efficiency, these results point toward a role of the AhR in the development of the visuo-motor system.

**Figure 6:**
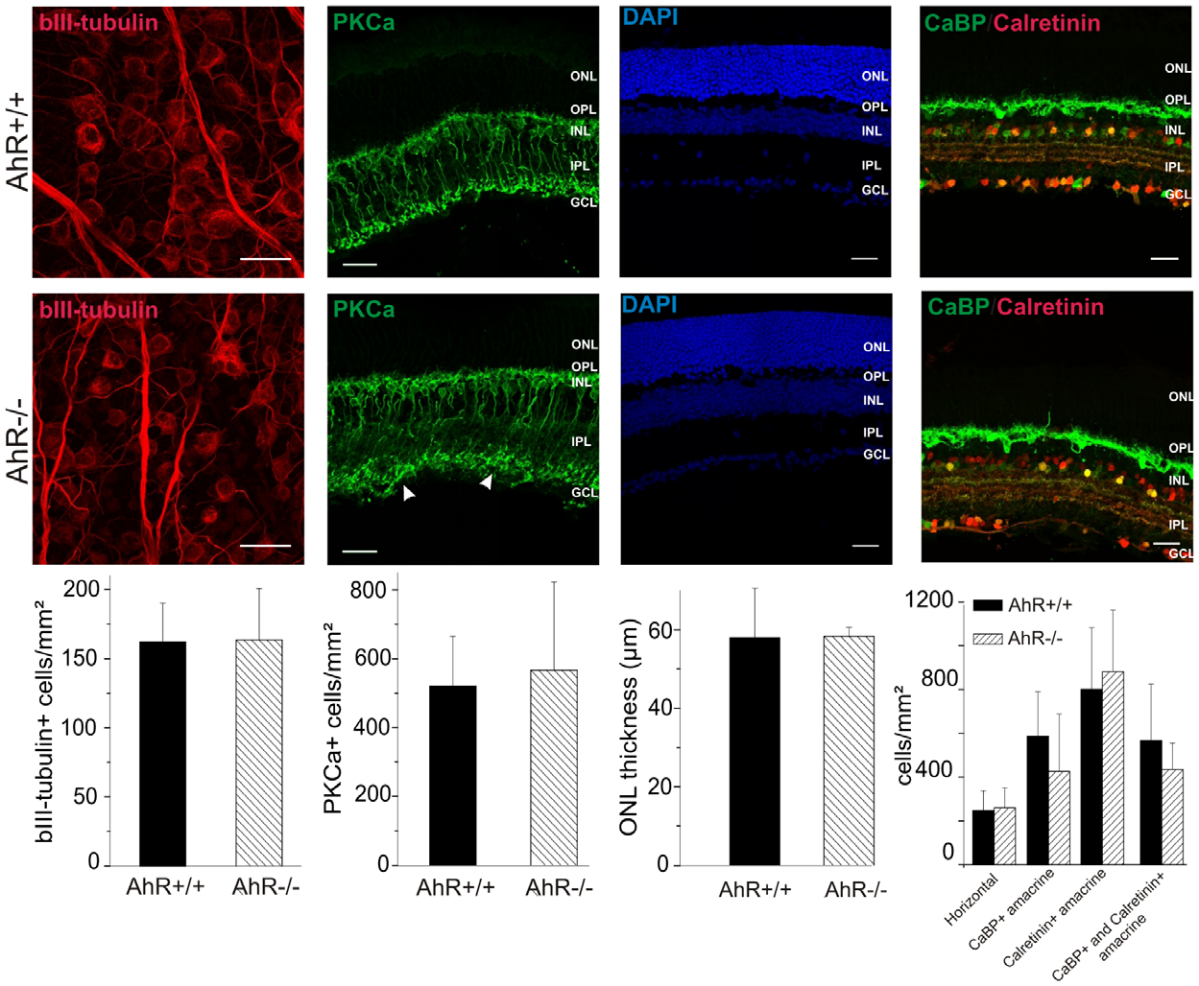
AhR−/− mice retinal cells are functional despite scattered bipolar to RCG connections. Immunohistochemistry experiments were performed in retina sections of adult AhR+/+ and AhR−/− mice. AhR+/+ and AhR−/− retina at P56 is represented. Retinal ganglion cells (RGC) are labeled with antibodies against ßIII-tubulin; bipolar cells are labeled with antibodies against PKCα (Protein Kinase Cα), nucleus is labeled with DAPI; amacrine and displaced amacrine cells are labeled with both antibodies against calbindin (CaBP)-D28k and calretinin. Horizontal cells are stained with antibodies against calbindin. The density of RGC, bipolar, amacrine and displaced amacrine, and horizontal cells are not significant different between AhR+/+ and AhR−/− mice (see quantifications at the bottom of the figure). The thickness of the ONL is the same between both genotypes. The white arrows in the bottom panel of the PKCa staining indicate a disorganization of the synapses between the bipolar cells and the RGCs in some AhR−/− mice. Scale bar  = 25 µm. ONL: Outer Nuclear Layer, INL: Inner Nuclear Layer, GCL: Ganglion Cells Layer, OPL: Outer Plexiform Layer, IPL: Inner Plexiform Layer.

To further address the cellular origin of the deficit, we investigated the histology of the retina in the AhR−/− mice using immunohistochemistry. We quantified the number of cells in the retina of adult AhR−/− mice (n = 4) and AhR+/+ mice (n = 4). Altogether, we found no quantitative difference between the number of retinal cells found in the AhR−/− and AhR+/+ mice (figure 6, bottom panels). However, we observed a clear disorganization of the synapses between the bipolar cells and the RGCs in most AhR−/− mice (3 out of 4; Figure 6, see the arrows in the bottom panel corresponding to the staining of PKCa in the AhR−/− mice); in the AhR +/+ mice, the distribution of the projections of the bipolar neurons onto the RCGs dendrites was found to be lamina-restricted (4 out of 4; Upper panel, PKCa staining) while these projections were scattered with the synaptic boutons of the bipolar cells disseminated throughout the IPL of the retina in some AhR−/− mice.

**Figure 7:**
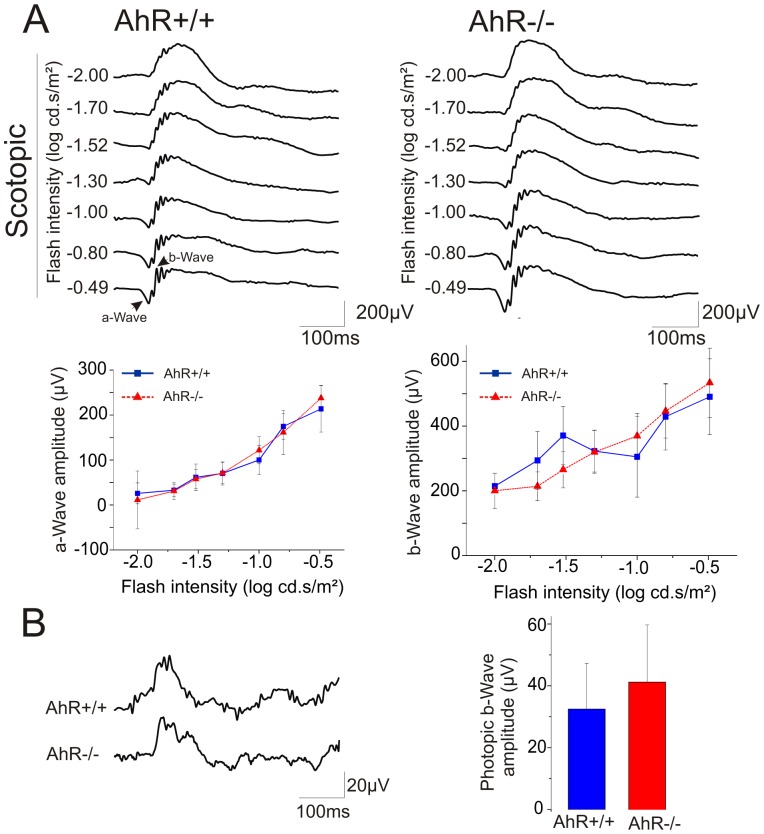
Electroretinograms are normal in AhR−/− mice. (A) Representative scotopic ERG at different light intensities (from −2 to −0.49 log cd.s/m^2^) in both AhR+/+ and AhR−/− mice. Quantifications of a-Wave and b-Wave amplitudes are represented in the lower panel. (B) Representative photopic ERG. The photopic response is obtained using a flash intensity to −0.49 log cd.s/m^2^ on light-adapted mice and measured for both mice. There are no significant differences between both genotypes in scotopic and photopic ERG. Blue lines correspond to the AhR+/+ mice, and red lines correspond to the AhR−/− mice.

To assess the function of the retinal cells, electrophysiological testing was performed using electroretinography. During a scotopic stimulation AhR+/+ response consisted of typical first negative a-wave followed by a positive b-wave (AhR+/+ mice on the left of figure 7A left traces). Responses observed in AhR−/− mice were similar to those of AHR+/+ (Figure 7A right traces). Quantification revealed no statistical difference between the two genotypes (Figure 7A bottom panels).

The photopic ERG wave amplitudes, reflecting cone function were also recorded after 5 minutes of light adaptation (Figure 7B). There was no significant difference of the photopic b-wave between the adult AhR−/− and AhR+/+ mice.

Altogether, our results demonstrate that the nystagmus observed in the AhR−/−mice is not related to a major histological or functional impairment of the retinal cells. However, decrease in optokinetic reflex, influence of light on the nystagmus, developmental expression of AhR in the retina and the observed disorganization of retinal cell layers all suggest that the nystagmus is likely of visual or visuo-motor origin.

### Exposure of Adult Mice to an Exogenous AhR Ligand, TCDD, does not Induce a Pendular Nystagmus

Finally, several recent reports suggest that exogenous AhR ligands might disrupt the endogenous function of the receptor. Sartor and colleagues reported in a hepatic cell line Hepa1c1c7 that the AhR binds different xenobiotic responsive elements with or without TCDD [25]. This phenomenon might be responsible for unpredicted toxicities. As a consequence, the disruption of the AhR functions through either the binding to exogenous ligands or genetic deletion could result in comparable phenotypes.

To test this hypothesis, we treated adult AhR+/+ mice (10 weeks) with TCDD, an exogenous ligand of the AhR, and measured several visuo-motor parameters as in the AhR−/− mice; we performed a subchronic treatment with 25 µg/kg of TCDD (or corn oil, as sham control), once a week during 5 weeks and daily observed the spontaneous eye movements (Figure S2). After 5 weeks of treatment, we did not observe any abnormal eyes movements, i.e no nystagmus was present, suggesting that in adult mice TCDD does not alter the putative physiological functions of the AhR related to the regulation of the gaze stabilizing homeostasis.

## Discussion and Conclusion

### Evidence for an Endogenous Role of AHR in CNS Development

Several studies investigated the effect of TCDD, the AhR ligand, on the brain development. TCDD exposure disrupts different cellular processes in neurons and glial cells such as apoptosis, proliferation and differentiation. For example, TCDD leads to an increase of apoptosis in granule neuron precursor cells [26] and in cortical neurons [6]. Moreover, using telencephalon tissues in E13.5 mice, Gohlke and colleagues suggested a disruption of the differentiation of GABAergic neurons in the ventral telencephalon in TCDD-treated WT mice and in non-treated AhR−/− mice [27].

Previous studies have suggested that the AhR plays a significant role in neuronal development and in neural functions in invertebrates. Interestingly, in *C*.*elegans*, the AhR plays a crucial role in the development of the fate of GABAergic neurons [10]. Based on these results, it is tempting to speculate that AHR endogenous functions in the CNS could have been conserved in vertebrates. Then, the binding of TCDD on the AhR could lead to a disruption of the endogenous functions of this receptor.

Very few studies have been performed using vertebrate models on the role of the AhR in the nervous system. It was suggested that the AhR could play an endogenous role in the neural biology [27]. Indeed, Lin et al., which used primary cortical neurons of rats, showed that a knockdown of AhR by siRNA experiments protects neurons against NMDA-mediated excitotoxicity [28]. The knockdown of the AhR induced a reduction of the NMDA-induced [Ca2+]_i_ influx due to a decreased expression of subunits of NMDA receptors. Still at the cellular level, the AhR also seems to be important for cell differentiation. The overexpression of the AhR in the murine Neuro2 cell line induces an outgrowth of the neuritis [29], which is also induced by treatment with TCDD, in granule neuroblast cells [8]. So far however, no study reported behavioural impairments in relation to AhR perturbation.

### Nystagmus: Comparison with Human Pathologies

In our study, we showed that the AhR−/− mice suffer from a horizontal pendular nystagmus whose molecular mechanisms are not yet characterized. In humans, congenital nystagmus (or as reported by the Committee for the classification of Eye Movement Abnormalities and Strabismus: infantile nystagmus syndrome, INS) is characterized by conjugated involuntary oscillations of both eyes, which appear at birth or during early infancy. INS as a prevalence of about 1/1000 to 1/6000 [30] is predominantly horizontal and often characterized by pendular waveforms in the 0.5–8 Hz range which can evolve with age in jerk movements. The nystagmus also increases with fixation attempt. The eye movement disorder we report in the AhR−/− mice shares many features with INS in humans. Hence, it appeared early in the animal development (<4weeks), was conjugated and predominantly horizontal. In addition as in humans, we report an evolution with age from pendular to jerk-like movements (Figure S1). Mouse nystagmus was also greater at light than in the dark, a symptom, which resembles INS during fixation. Interestingly, zebrafish and sheepdogs were for long the only animal models used in INS research but their nystagmus is not persistent under obscurity [31]. Recently, albino mice were proposed as a model for INS [32]. Melanin synthesis disorders have long been linked to visual and visuo-motor impairments and undoubtedly constitute an interesting model for INS in albinos patients (∼25% of INS patients; [33]). The AhR−/− mouse is complementary to these models and shows some unique features, which might be relevant to perform additional works that cannot be realized with other albino strains of mice or other animal models. Given the many common features between the AhR mouse and human INS, it would be interesting to characterize if the other strains of AhR−/− mice generated independently (whose phenotypes sometimes diverge) [4], [5], display a nystagmus.

### Structure and Systems Potentially Implicated in Nystagmus of Visual or Visuo-motor Origin

The functional deficit observed in the optokinetic reflex suggests that the pendular nystagmus observed in AhR−/− is likely of visuo-motor origin. The circuitry of the optokinetic system involves the retina, the afferent visual pathway, the accessory optic system, inferior olive and cerebellum, many brainstem oculomotor-related nuclei, oculomotor nerves (III et VI for horizontal movement) and the extra-ocular muscles [34].

Congenital nystagmus is often associated to a decrease of visual acuity or retinal impairments. For instance, it may be due to a loss of photoreceptors like in the Leber’s congenital amaurosis (LCA) [35]. Interestingly, mutations of AIPL1 (AhR interacting protein like-1), which shares 49% identity and 69% similarity with the human AIP (AhR interacting protein), was identified as a cause for this inherited disease [36]. Our results suggest that AhR−/− mice do not suffer from a loss of retinal cells, or of photoreceptor as in LCA. We have however identified in some AhR−/− mice a disorganization of lamina-restricted connections between bipolar axons and RGCs dendrites, but ERG didn’t reveal any functional deficit in the retina, suggesting that the basic physiology of the retinal cells and circuitry might be preserved. Whether this disorganization of retina cell layers relates to the transitory expression of the AhR during development, the origin of the optokinetic reflex deficit and congenital nystagmus is currently unknown and will be the topic of future investigations.

Finally, AhR−/− mice had a transparent cornea and lens such that no obvious anatomical alteration of the eye was observed. Specific functional test of visual acuity in AhR−/− mice will be needed to distinguish between a purely visual or visuo-motor origin of the nystagmus. Hence, congenital nystagmus is most probably multifactorial, and other defects downstream of the retina could be implicated in both OKR deficit and nystagmus. The electrophysiological investigation of structures such as the NOT (Nucleus Optic Tract), inferior olive nucleus, abducens and oculomotor nuclei, will likely be needed in order to functionally trace the neuronal origin(s) of the spontaneous nystagmus in the AhR−/− mice.

### Toxicological Activation of AHR and Myelination

Nystagmus are often found in myelin disorders [37], [38]. It was recently demonstrated that the treatment of rats by TCDD during gestation altered the expression of genes involved in the myelin, suggesting that activation of the AhR by exogenous ligands might lead to disruption of myelin homeostasis [39]. Future experiments focusing on the myelination of the AhR−/− brain will explore this hypothesis.

Finally, treatment with TCDD might be also interesting to test the hypothesis of an endogenous disruption of the normal functions of the receptor. We already demonstrated that AhR+/+ mice treated by high doses of TCDD during 6 weeks do not exhibit an ocular instability. However, It will be interesting to treat pregnant mice to assess the consequences of TCDD exposure on the developing brain and grown behavior of the pups.

### Conclusion

In conclusion, our study demonstrates a new endogenous role of the AhR in the homeostasis of the developing nervous system. To our knowledge, no mouse model of congenital nystagmus has been reported so far. Future work on the AhR−/− mice will help to better understand the developmental mechanisms underlying congenital nystagmus.

## Supporting Information

Figure S1 **Evolution of the nystagmus throughout mice aging.** Position of the eyes in the horizontal plan (EHP) of two AhR−/− mice at different ages (10 and 23 weeks for mice 1; 24 and 38 for mice 2). The figure presents discontinuous time, as indicated by the vertical lines. EHP, Eye Horizontal position.
(TIF)

Figure S2 **TCDD treatment of adult AhR+/+ mice did not affect gaze stability.** Adult AhR+/+ mice were treated by 25 µg/kg of TCDD at day 0, 7, 14, 21, 28. The mice were monitored at day 0, 13, 27, 34 (respectively D0, D13, D27, D34). Positions of the eyes in horizontal (EHp) plan in the absence of head movements in the dark are presented. The AhR +/+ mice treated by TCDD did not exhibit a horizontal pendular nystagmus. Note D13 trace shows discontinuous time, as indicated by the vertical line. EHp, Eye Horizontal position.
(TIF)
