# Supplementary figures and images for: Oculomotor Deficits in Aryl Hydrocarbon Receptor Null Mouse
Source: PLoS One. 2013 Jan 3;8(1):e53520. doi: 10.1371/journal.pone.0053520 (PMC3536739; doi:10.1371/journal.pone.0053520)

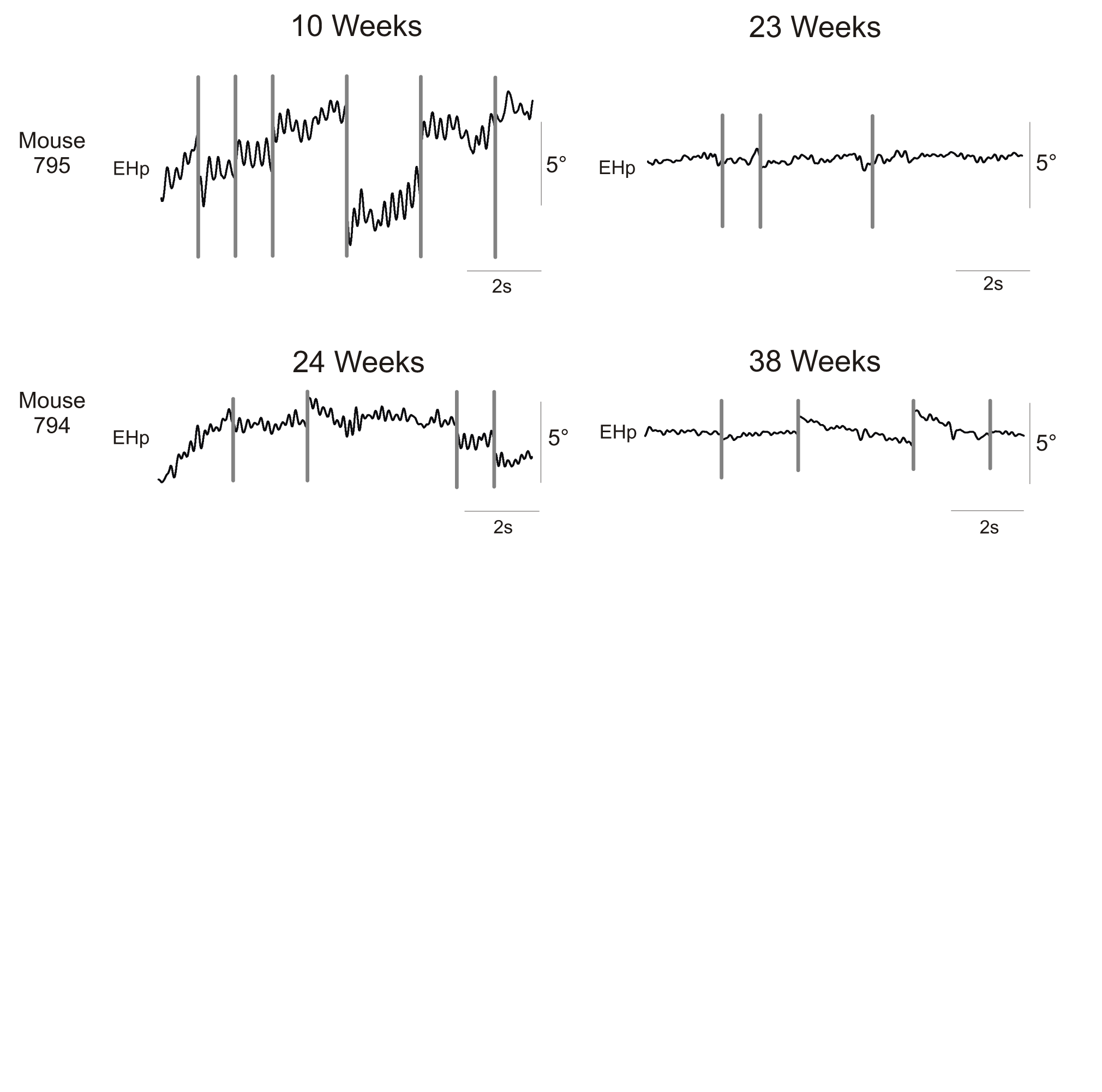

Supplement: Figure S1 — Evolution of the nystagmus throughout mice aging. Position of the eyes in the horizontal plan (EHP) of two AhR−/− mice at different ages (10 and 23 weeks for mice 1; 24 and 38 for mice 2). The figure presents discontinuous time, as indicated by the vertical lines. EHP, Eye Horizontal position. (TIF) [file pone.0053520.s001.tif]

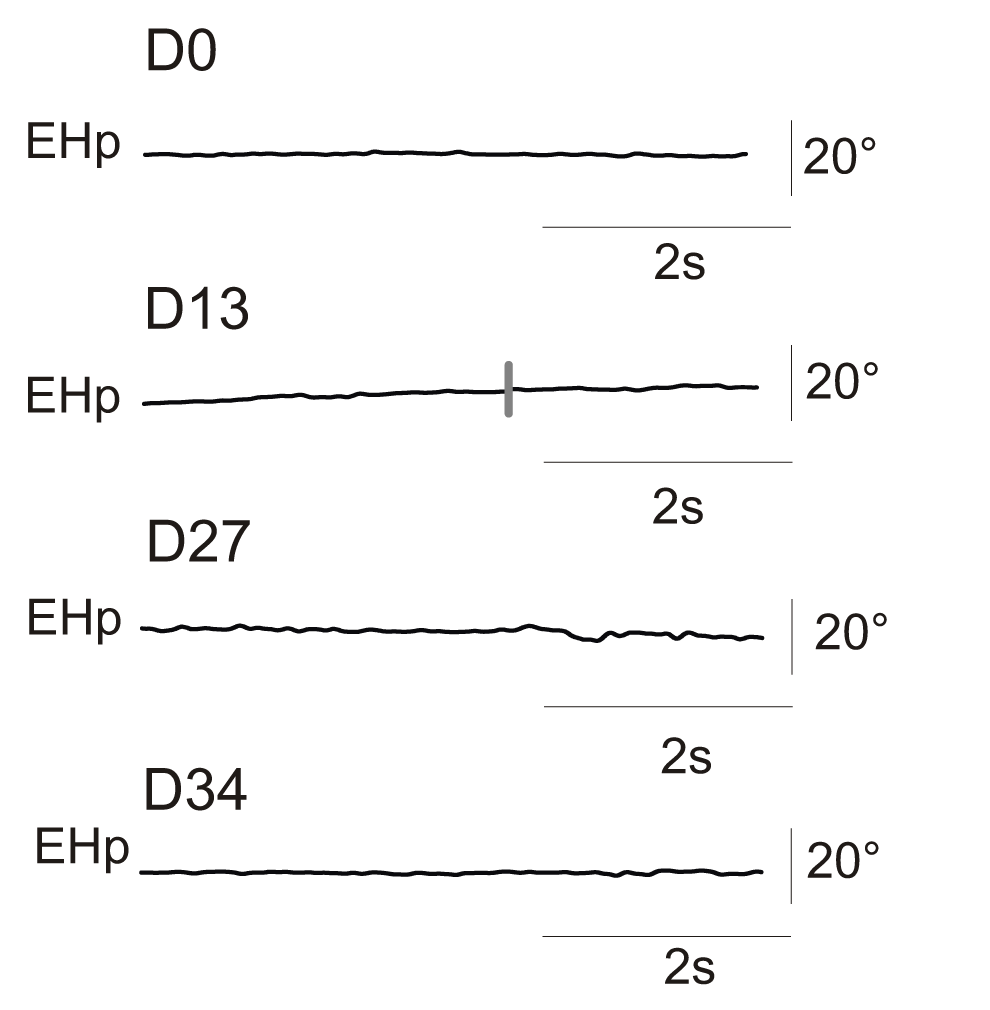

Supplement: Figure S2 — TCDD treatment of adult AhR+/+ mice did not affect gaze stability. Adult AhR+/+ mice were treated by 25 µg/kg of TCDD at day 0, 7, 14, 21, 28. The mice were monitored at day 0, 13, 27, 34 (respectively D0, D13, D27, D34). Positions of the eyes in horizontal (EHp) plan in the absence of head movements in the dark are presented. The AhR +/+ mice treated by TCDD did not exhibit a horizontal pendular nystagmus. Note D13 trace shows discontinuous time, as indicated by the vertical line. EHp, Eye Horizontal position. (TIF) [file pone.0053520.s002.tif]
